# MALDI MSI and Raman Spectroscopy Application in the Analysis of the Structural Components and Flavonoids in *Brassica napus* Stem

**DOI:** 10.3390/metabo13060687

**Published:** 2023-05-25

**Authors:** Mikolaj Krysa, Katarzyna Susniak, Adrianna Kubas, Dominika Kidaj, Anna Sroka-Bartnicka

**Affiliations:** 1 Independent Unit of Spectroscopy and Chemical Imaging, Faculty of Biomedicine, Medical University of Lublin, Chodźki 4a Street, 20-093 Lublin, Poland; mikolajkrysa@umlub.pl (M.K.); ada.kubas.22@gmail.com (A.K.); 2 Department of Pharmaceutical Microbiology, Faculty of Pharmacy, Medical University of Lublin, Chodźki Street 1, 20-093 Lublin, Poland; katarzyna.susniak@umlub.pl; 3 Department of Genetics and Microbiology, Institute of Biological Sciences, Maria Curie-Sklodowska University, Akademicka 19, 20-033 Lublin, Poland; dominika.kidaj@mail.umcs.pl

**Keywords:** *Brassica napus*, rapeseed, Nod factors, biofertilizer, MALDI mass spectrometry imaging, Raman spectroscopy

## Abstract

Nod factors among the signaling molecules produced by rhizobia in response to flavonoids to induce root nodule formation in the legumes. It is, however, hypothesized that they might increase the yield and positively impact the growth of non-legumes. To evaluate this statement, rapeseed treated with Nod factor-based biofertilizers were cultivated, their stems was collected, and the metabolic changes were investigated using Raman spectroscopy and MALDI mass spectrometry imaging. Biofertilizer proved to increase the concentration of lignin in the cortex, as well as hemicellulose, pectin, and cellulose in the pith. Moreover, the concentration of quercetin derivatives and kaempferol derivatives increased, while the concentration of isorhamnetin dihexoside decreased. The increase in the concentration of the structural components in the stem might therefore increase the lodging resistance, while the increase in concentration of the flavonoids might increase their resistance to fungal infection and herbivorous insects.

## 1. Introduction

Rapeseed (*Brassica napus* L.) is a crucial oil plant worldwide [1]. Its seeds are a primary source of edible oil and biodiesel [2]. During the oil extraction process, rapeseed meal is often produced as a protein-rich by-product [3].

One of the main factors that negatively affect rapeseed yield is lodging, which occurs when the stem loses its rigidity and strength due to a lower concentration of structural components such as cellulose, hemicellulose, and lignin [4,5]. The lodging resistance can be increased by regulating the density of plants, regulating the nitrogen fertilization rate, and by the addition of external substances. A too-low density of the plants causes low rapeseed yield per hectare (but high lodging resistance), whereas too-high plant density leads to increased resource competition and therefore higher stem growth with lower amounts of structural components, which leads to lower lodging resistance [6,7]. Optimization of nitrogen fertilizer use is also important, because too-low fertilization may lead to nitrogen shortage in the soil and lower yield; while-too high nitrogen fertilization might lead to higher production costs, environmental pollution, and decreased concentration of the structural metabolites [4,5,8,9].

Other factors that might decrease the rapeseed yield are herbivores (mainly insects) and fungi infections. The infections and losses due to herbivores are even more severe in monocultures, which are currently dominant in terms of plant cultivation [10]. A common practice to reduce costs of yield losses is the use of insecticides and fungicides. Despite being very effective, the overuse of them is costly, negatively impacting the environment and human health [11,12]. Plants (including rapeseed), however, do have innate defence systems to protect against both herbivores and infections. One of those mechanisms is the production of flavonoids [13]. The protection against herbivores is due to their acting as the antifeedants [14] and feeding deterrents [15], which results in decreased pest survival [16]. Flavonoids also crosslink and inhibit enzymes and form physical barriers to protect against fungal infections [17].

Due to the large area used for rapeseed cultivation, there is a growing need for cheap, efficient, and environmentally friendly fertilizers. Goods candidates for such a role are Nod factors. These lipo-chitooligosaccharides are produced by bacteria of *Rhizobium* genus in response to the flavonoids that they detect in the soil. The flavonoids (such as genistein or naringenin) are produced by the legume plants and are released to the soil to attract the *Rhizobium* bacteria. The bacteria release Nod factors, which signal to the plants that these bacteria are nearby, and thus to start the formation of the root nodules for rhizobia to settle. The plants will provide a carbon source, and rhizobia will provide a nitrogen source that was fixed from the air [18]. As a result, less nitrogen fertilizers are needed during legume cultivation. Due to this effect, the hypothesis was formed that the addition of the external Nod factors would induce the higher growth and better development of legumes; indeed, the addition of external Nod factors turned out to induce the growth and development of the legume plants [19]. Due to the inability to form the root nodules in non-legumes, Nod factors were not considered potential growth-promoting factors in the non-symbiotic plants. In several studies, however, the effects of purified Nod factors were evaluated on such plants, showing that Nod factors might increase their growth and development [20,21], including their photosynthetic rate [22]. The possible mechanism of action of Nod factors on non-legume plants was proposed by Liang et al.—they showed that microbe-associated molecular pattern triggered immunity and the innate immune response of the plant is suppressed by Nod factors [23]; however, the exact mechanism of action of Nod factors on the non-host plants is not yet fully understood.

To better understand the effect and mechanism by which Nod factors affect non-symbiotic eudicots, rapeseed stems whose seeds were previously inoculated in the fractionated n-butanol phase of *Rhizobium leguminosarum* bv. *Viciae* bacterial supernatant (RLN) or in the fractionated water phase of *Rhizobium leguminosarum* bv. *Viciae* bacterial supernatant (K60) were investigated. The methods used for this purpose were Raman spectroscopic imaging and matrix-assisted laser desorption/ionization mass spectrometric imaging (MALDI MSI). This research aims to better understand the potential of Nod factors as a cheap, efficient, and environmentally friendly fertilizer for large-scale rapeseed cultivation.

## 2. Materials and Methods

### 2.1. Biofertilizer Preparation

The biofertilizer was prepared as described previously [19] and in accord with the patent [PL240045] (https://ewyszukiwarka.pue.uprp.gov.pl/search/pwp-details/P.433315?lng=en, accessed on 1 April 2023). Briefly, the *Rhizobium leguminosarum* bv. *Viciae* bacteria were induced with naringenin and the bacterial supernatant was fractionated twice. First, the n-butanol extraction was performed (using water as a second solvent); then, the fractionation on an SPE column was performed. Both the n-butanol fraction (which contains Nod factors) and water fraction (which does not contain Nod factors) were subjected to the second fractionation. Then, the 60% acetonitrile fraction was collected (from both water and n-butanol fractions) and their concentration was increased through evaporation. 

### 2.2. Rape Cultivation

The rapeseeds were soaked for 30 min in either a fractionated solution of n-butanol fraction containing Nod factors (RLN samples), a fractionated solution of water fraction that does not contain Nod factors (K60 samples) (the preparation of these biofertilizers is described in a Section 2.1), or purified water (Control samples). Seeds (seven seeds per pot) were then sown in garden soils–and–perlite mixture (in a 1:1:1 volume ratio). The plants were grown in Maria Curie-Skłodowska University’s greenhouse, were watered three times a week, and collected after 42 days of cultivation. Then, the whole plants were flash-frozen in liquid nitrogen and kept at −70 °C until used.

### 2.3. Raman Sample Preparation and Data Acquisition

Three randomly chosen stem samples from each group (n = 3 per group) were cut on the cryo-microtome on the 20 µm slices (Leica CM 1950, Leica Biosystems, Wetzlar, Germany) in −20 °C and thaw-mounted on the aluminium coated slides. The glass slides were then dried using a desiccator and the Raman spectroscopic imaging was carried out in the cortex and the pith’s stem. The imaging was conducted with a 1.5 × 1.5 µm step size and a single map consisted of 49 or 64 points. The Raman spectrometer (DXR Raman confocal microscope, Thermo Scientific, Waltham, MA, USA) was equipped with a 780 nm laser with a maximum laser power of 24 mW. The aperture size was 50 µm pinhole. The number of scans per single spectra was 10 and the length of one scan was 15 s. The spectra were recorded in the 210–2000 cm^−1^ range.

### 2.4. Raman Spectroscopic Imaging Data Curation

Since the Raman spectroscopic imaging data did not provide sufficient spatial distribution differences between the groups, the imaging spectra were treated as regular spectra. Raman spectra were baseline corrected using the rubber band method [24] using Quasar software (v. 1.5.0.) with a spectroscopy add-on [25]. The spectra were then vector normalized, principal component analysis (PCA) was performed, and the principal components (PC) that separated the spectra best were chosen for the analysis. For this purpose, loading plots with a threshold over 0.6 annotated were prepared from the chosen PCs. The crossing of the threshold and the width of the region over 10 cm^−1^ was considered sufficient to consider the region significant. The significant regions were then plotted on the spectra with a 95% confidence interval in the range −100 and +100 cm^−1^ from the centre of the region. Vector normalization, PCA, loading plot preparation, and average spectra plotting were conducted using Python programming language (v. 3.8.10) with Spyder IDE (v. 5.3.0) and packages: Scikit-learn, matplotlib, and seaborn [26,27,28]. The code used for this purpose is shown in Supplementary Materials (Code S1, Code S2).

### 2.5. Sample Preparation for MALDI MS Imaging

Whole plants of *Brassica napus* were flash-frozen in a liquid nitrogen and stored at −70 °C. Stems from each group were cryosectioned into 20 µm slices at −20 °C using Leica CM1950 cryomicrotome (Leica Biosystems, Wetzlar, Germany). Six to nine slices were obtained from each group and attached to no-frost standard microscopic slides. The slices were embedded in a 0.3 µm drop of a MALDI matrix for better adhesion to the glass slide and to create the nucleation sites. The tissue was afterward covered with multiple coats of a matrix using an airbrush. The matrix used for the analysis was α-cyano-4-hydroxycinnamic acid (CHCA, 20 mg/mL in a solution of 70% acetonitrile in 30% water with an addition of 0.1% TFA, Merck, Darmstadt, Germany).

### 2.6. MALDI Mass Spectrometry Imaging

MALDI MS imaging was carried out using MALDI QTOF high-definition mass spectrometer (Synapt G2-*Si* HDMS, Waters Corporation, Milford, MA, USA) equipped with an Nd:YAG laser. Imaging analyses were acquired with settings as follows: positive ionization mode; mass range 100–1500 *m*/*z* range; laser power—250 eV; reception rate—1000 Hz; laser step size—55 µm × 55 µm (in the XY axis). Two separate imaging analyses were performed for slices from each group. Images were obtained and analyzed using HD Imaging software (ver. 4.1, Waters Corporation, Wilmslow, UK). Generated maps were normalized to the total ion current. Identification of the compounds was based on databases such as MassBank of North America and PubChem.

## 3. Results

### 3.1. The Comparison of Different Groups in the Cortex Raman Spectra

**Figure 1:**
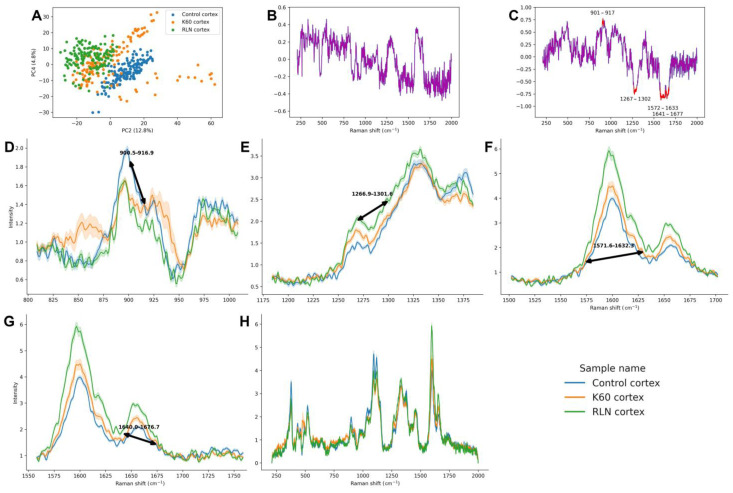
PCA, loading plots, and mean spectra (with 95% confidence interval) of regions that were over the 0.6 (or below −0.6) threshold in the loading plot of the rapeseed stem cortex after different treatments. In the PCA, PC2 score is plotted against the PC4 score (**A**), and the percentage of described variation is shown next to the axes. The first loading plot (**B**) shows the PC4 loading plot without the regions crossing the threshold of over 0.6 or below −0.6. The second loading plot (**C**) shows the PC2 loading plot without the regions crossing the threshold of over 0.6 or below −0.6, which are considered significant. The regions that cross the threshold are: 901–917 cm^−1^ (spectra of this region shown on panel (**D**)); 1267–1302 cm^−1^ (panel (**E**)); 1572–1633 cm^−1^ (panel (**F**)); 1641–1677 cm^−1^ (panel (**G**)). All regions crossing the threshold are characteristic to lignin. The full spectra are shown on panel (**H**).

Raman PCA of the cortex spectra showed that PC2 plotted against PC4 provided the most sufficient separations between the RLN, K60, and control samples (Figure 1A) (other cortex PCA plots are shown in the Supplementary Materials, Figures S2–S7). Both PC2 and PC4 were needed to efficiently separate the groups; therefore, the loading plots with 0.6 threshold crossing marked were analyzed. Unfortunately, in the PC4 loading plot (Figure 1B), none of the bands crossed the threshold (due to too small contribution of any band to PC4); therefore, only the PC2 loading plot (Figure 1C) was analyzed.

The first significant differentiation in the PC2 loadings is in the region 901–917 cm^−1^, which is a part of lignin CCH wagging vibration (Table 1). This band has a higher area in the control cortex, while in the K60 and RLN cortices, it presented similar, smaller area (Figure 1D). Another significantly differentiating region is the one between 1267–1302 cm^−1^, which is a part of the aryl–O of aryl OH and aryl O–CH_3_ and guaiacyl ring (with C=O group) mode region characteristic of lignin. It has the highest area in the RLN cortex samples; the intermediate is presented by K60 samples; and the lowest by control ones (Figure 1E). Similar changes are presented by two bands in the regions 1572–1633 and 1641–1677 cm^−1^, which arise due to the symmetric aryl ring stretching of lignin and the ring C=C stretching of coniferyl alcohol and C=O stretching of coniferaldehyde in lignin, respectively (Figure 1F,G. respectively). They both have the highest area in the RLN sample, lowest in the control, and intermediate in the K60 sample. Mean spectra in the whole range (210–2000 cm^−1^) of the cortex are presented in Figure 1H.

### 3.2. The Comparison of Different Groups in the Pith Raman Spectra

**Figure 2:**
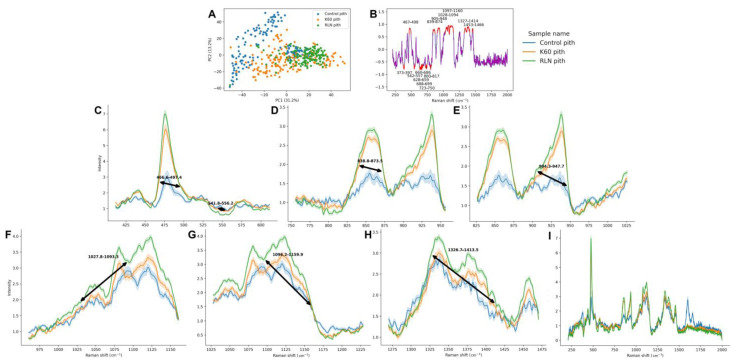
PCA, loading plot, and mean spectra (with 95% confidence interval) of regions that were over the 0.6 (or below −0.6) threshold in the loading plot of the rapeseed stem pith after different treatments. In the PCA, PC1 score is plotted against the PC2 score (**A**), and the percentage of described variation is shown next to the axes. Loading plot (**B**) shows the PC1 loading plot without the regions crossing the threshold of over 0.6 or below −0.6, which are considered significant. The regions that cross the threshold are as follows: 467–498 cm^−1^ (spectra of this region showed on panel (**C**)—hemicellulose region); 839–874 cm^−1^ (panel (**D**)—pectin region); 905–948 cm^−1^ (panel (**E**)—lignin region); 1028–1094 cm^−1^ (panel (**F**)—cellulose region); 1097–1160 cm^−1^ (panel (**G**)—cellulose region); 1327–1414 cm^−1^ (panel (**H**)—cellulose region). The full spectra are shown on panel (**I**).

PCA of the pith spectra showed that the best differentiation occurred due to the PC1 score. The separation of the control pith spectra was quite sufficient; however, the separation between the RLN and K60 samples was not so clear (Figure 2A) (other pith PCA plots are shown in the Supplementary Materials, Figures S8–S12). Due to the fact that the most separation between the samples occurred due to the PC1 score, the PC1 loading plot was analyzed (Figure 2B). PC1 loadings presented 16 bands that had contributed to the separation (bands that were over the 0.6 or below −0.6 threshold); therefore, only the bands that presented high differences in the areas were analyzed. The rest were shown in the Supplementary Material for clarity (Figures S13–S16).

All of the analyzed bands presented similar changes—the highest area is presented by RLN pith spectra, slightly lower is in K60, and the lowest is in the control spectra. The first band was in a 467–498 cm^−1^ range and is attributed to composite bending vibrations involving the C6 position of hemicellulose (Figure 2C). Next is in the 839–874 cm^−1^ range and is occurring due to COC skeletal vibrations of pectin (Figure 2D). The band in the 905–948 cm^−1^ range characteristic for CCH wagging of lignin also presented similar changes (Figure 2E), as well as two bands in the 1028–1094 and 1097–1160 cm^−1^ regions, both occurring due to CC and CO stretching and HCC and HCO bending of cellulose (Figure 2F,G, respectively). The last differentiating band with similar changes was in the 1327–1414 cm^−1^ range, which arises because of HCC and HCO bending and HCC, HOC, and HCO bending of cellulose (Figure 2H). The mean spectra in the whole range (210–2000 cm^−1^) of the pith are presented in Figure 2I.

Since the comparison between the pith and the cortex is beyond the scope of this article, it was presented in Supplementary Materials (Section S1, citations [35,36,37,38]).

### 3.3. The Distribution of the Flavonoids in the Rapeseed Stem

**Figure 3:**
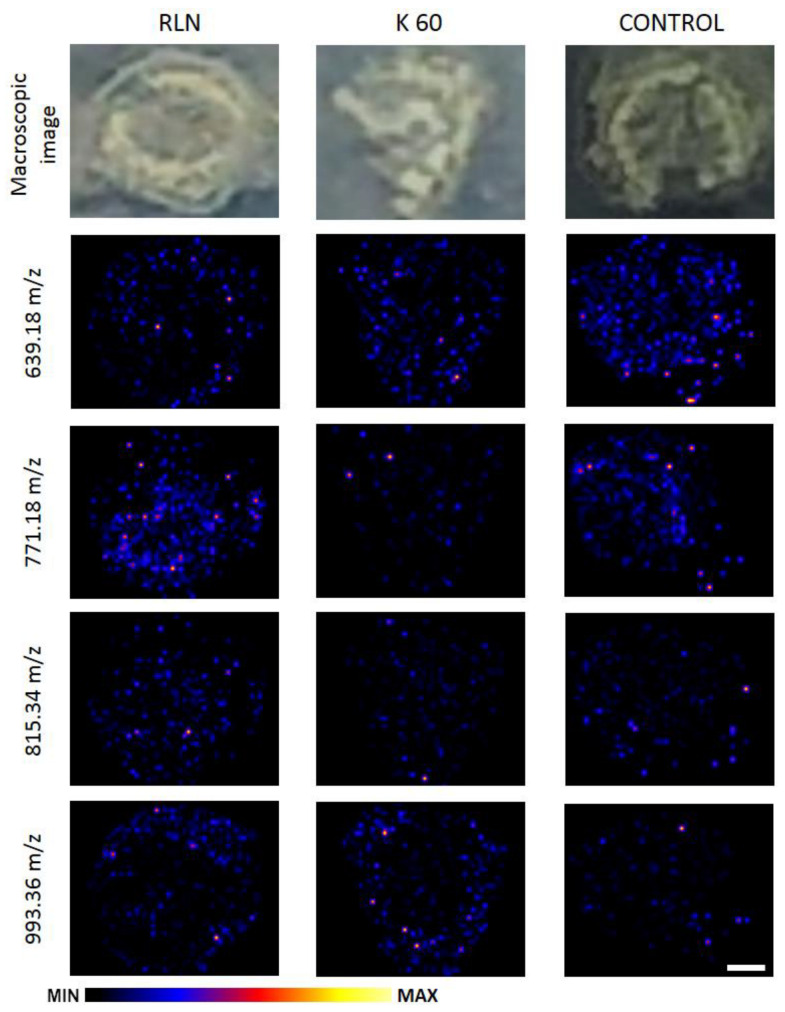
Macroscopic image of *Brassica napus* stems and chemical maps of selected metabolites (639.18 *m*/*z*—isorhamnetin dihexoside; 771.18 *m*/*z*—quercetin trihexoside; 815.34—kaempferol sinapoyl dihexoside; 996.36—quercetin sinapoyl trihexoside) obtained by MALDI MSI. The bar represents 0.5 mm.

Stems of plants from each group were analyzed using MALDI MSI. Several compounds from a large group of flavonoids present in *B. napus* were selected, and the chemical maps of their distribution in the tissues are presented in Figure 3. Derivatives of the three most frequently occurring flavonoids (quercetin, kaempferol, and isorhamnetin) were identified by comparison of MS data with databases and literature. Based on Farag et al. [39], signal 639.18 *m*/*z* comes from isorhamnetin derivative—isorhamnetin dihexoside. Its presence in the stem tissue is slightly less intense after treatment with any of preparations in the comparison to the control. Signals 771.18 *m*/*z* and 993.36 *m*/*z* were assigned to quercetin derivatives—quercetin trihexoside and quercetin sinapoyl trihexoside respectively. The presence of both of them is more significant in the tissues after treatment with the preparations compared to the control. The 771.18 *m*/*z* signal is detected with higher intensity in the tissue after the application of RLN biofertilizer than in the tissue after application of K 60 fraction, where its presence is minimal. Meanwhile, the signal presence of the 993.36 *m*/*z* signal in the whole tissue is similar in both RLN and K60 groups. The Third featured flavonoid is kaempferol and the signal 815.34 *m*/*z* was assigned to its derivative—kaempferol sinapoyl dihexoside. It is present evenly in the tissue of plants treated with RLN fraction; whereas it has a minor presence in plants treated with K60 fraction and in the control. 

## 4. Discussion

There are a couple of ways to enhance lodging resistance in plants (mentioned in the introduction section), and one such method involves using external substances. Specifically, triazole-type compounds have been identified as a group of substances that can inhibit the growth of rapeseed by acting as gibberellin synthesis inhibitors. This leads to a higher stem density and improved lodging resistance [40,41]. Similar studies were conducted on maize, where growth regulators were used. Liu et al. showed an increase in the concentration of lignin, cellulose, and hemicellulose in the stem [4]. Our findings suggest that Nod factor-based biofertilizers might also be effective in increasing lodging resistance in the rapeseed plants through a similar effect to that presented by Liu et al.—an increase in the lignification in the cortex and an increase in the concentration of hemicellulose, pectins, and cellulose in the pith of the stem.

While it is generally true that higher amounts of lignin, cellulose, hemicellulose, and pectins are beneficial for increased grain yield and lodging resistance, there are downsides to consider. These compounds can negatively impact the quality of rapeseed meal [42]; therefore. Nod factor-based biofertilizers should be used with caution for this purpose.

Since flavonoids may act as innate insecticides and fungicides, there have been some successful attempts to increase their concentration in plants to improve the plants’ resistance to herbivores and fungi. One such attempt was made by Skadhauge et al., who showed that mutant barley was particularly resistant to *Fusarium* infection [17]. It turned out that this high resistance was the effect of a high accumulation of dihydroquercetin, which resulted in the inability of fungi to infect the plant. Similar studies were conducted by Mallikarjuna et al. [43], who showed a species of groundnut resistant to tobacco armyworm (*Spodoptera litura*). The insecticide effect was mainly due to high quercetin concentration (similar to the studies of Skadhauge et al.). The determination of the level of flavonoids, however, is not limited to genetic factors. Their concentration might be modulated by environmental factors such as propagation regime [44], UV-B radiation [45,46], or the addition of Nod factors (as we showed in this report). Our results revealed that the amount of quercetin derivatives and kaempferol derivative is higher in plants treated with biofertilizer than in the control plants, while the concentration of isorhamnetin dihexoside decreased. The increase in the quercetin derivatives is especially encouraging because it acts as both insecticide and fungicide [17,43].

Isorhamnetin-dihexoside, quercetin-trihexoside, kaempferol-sinapoyl-dihexoside, and quercetin-sinapoyl-trihexoside, which we found in the rapeseed stem, are flavonols; therefore, they have similar biosynthetic pathway: products of the polypropanoid pathway—4-coumaroyl-CoA and malonyl-CoA—are converted to naringenin chalcone by chalcone synthase. This is then converted to naringenin by chalcone isomerase. Naringenin is then converted to dihydrokaempferol with flavanone 3-hydroxylase. Then, the pathway splits into two: the pathway to synthesize kaempferol derivatives; and the pathway to synthesize both quercetin and isorhamnetin derivatives. To synthetize kaempferol derivatives, the dihydrokaempferol is converted by flavonol synthase to kaempferol, which is then converted to keapferol derivatives (e.g., kaempferol-sinapoyl-dihexoside) by uridine diphosphate glycosyltransferase. To synthesize quercetin and isorhamnetin derivatives, dihydrokaempferol is converted to dihydroquercetin by flavonoid 3′-hydroxylase. Dohydroquercetin is then converted by flavonol synthase to quercetin, which is afterward converted to either quercetin derivatives (e.g., quercetin-trihexoside or quercetin-sinapoyl-trihexoside) by uridine diphosphate glycosyltransferase or to isorhamnetin by *O*-methyl transferase. Isorhamnetin is then converted to isorhamnetin derivatives (such as isorhamnetin-dihexoside), again by uridine diphosphate glycosyltransferase [47]. The increase in the concentration of quercetin and kaempferol derivatives presented mainly by Nod factor-based biofertilizer (RLN)—and, to a lower extent, by K60—might be due to an increase in the concentration of the flavonol synthase enzyme or any upstream enzyme (except flavonoid 3′-hydroxylase). The decrease in the concentration of isorhamnetin derivatives might be due to the decrease in the concentration of *O*-methyl transferase.

It is important to note that our findings on increased resistance to lodging and pests should be taken with caution. We did not directly evaluate stem endurance or the plant’s resistance to pests. Rather, we observed the metabolic changes that are associated with those effects, based on existing literature. Further research is necessary to confirm these findings.

## 5. Conclusions

Overall, Nod factor-based purified biofertilizer induces higher lignification of the rape cortex and increases the concentration of hemicellulose, pectin, and cellulose in the pith, which might improve the stem endurance and therefore increase the lodging resistance. Moreover, it increases the concentration of flavonoids, which might increase the resistance of the crops to herbivores and fungi infection. Purified water fraction of the fertilizer also seems to increase these metabolites, albeit to a lower extent. Application of purified Nod factor-based biofertilizer might therefore be a way to indirectly increase the yield of the rapeseeds in an environmentally friendly and cost-effective way. Moreover, MALDI MSI and Raman spectroscopy were proven to be complementary and universal techniques in the analysis of plant metabolites.

## 6. Patents

Patents resulting from this work: PL240045 (https://ewyszukiwarka.pue.uprp.gov.pl/search/pwp-details/P.433315?lng=en, accessed on 1 April 2023)

## Figures and Tables

**Table 1:** Characteristic bands found in the plant stem using Raman spectroscopy.

Raman Shift [cm^−1^]	Source	Functional Group	Literature
~1650	Lignin	Ring C=C stretching of coniferyl alcohol, C=O stretching of coniferaldehyde	[29,30]
~1600	Lignin	Symmetric aryl ring stretching	[29,30]
~1462	Lignin and cellulose	HCH HOC bending	[31]
~1393	Lignin	OH bending	[29]
~1376	Cellulose	HCC, HOC, and HCO bending	[31]
~1333	Cellulose	HCC and HCO bending	[31]
~1274	Lignin	Aryl–O of aryl OH and aryl O–CH_3_; guaiacyl ring (with C=O group) mode	[31]
1178–978	Cellulose	CC and CO stretching mainly + HCC, HCO bending	[29,30]
~927	Lignin	CCH wagging	[32]
860–825	Pectin	COC skeletal vibrations	[33]
515–475	Hemicellulose	Composite bending vibrations involving the C6 position	[33]
~436	Cellulose	CCO ring stretching	[34]
300–400	Cellulose (crystallinity)	Out-of-plane bending of C–O–C glycosidic bonds and OH groups coupled with simultaneous expansion and contraction in all rings	[29,30]

## Data Availability

The data presented in this study are openly available in Mendeley Data at https://doi.org/10.17632/v49khd5kr3.1 (accessed on 1 April 2023).

## Supplementary Materials

The following supporting information can be downloaded at: https://www.mdpi.com/article/10.3390/metabo13060687/s1, Code S1: Python code adding names to the samples, performing vector normalization, performing principal components analysis, and creating loading plots; Code S2: Python code creating the spectra with 95% confidence interval for mean from the whole spectral range and from the parts of the spectra that cross the threshold; Figure S1: PCA, loading plot and mean spectra (with 95% confidence interval) of regions that were above the 0.6 (or below −0.6) threshold in the loading plot. In the PCA PC1 score is plotted against the PC2 score (A) and the percentage of described variation is shown next to the axes. The loading plot (B) shows the PC2 loading plot with the regions crossing the threshold of above 0.6 or below −0.6, which are considered significant. The regions that cross the threshold are: 370–391 cm^−1^ (spectra of this region showed on panel C—cellulose region), 436–448 cm^−1^ (panel C—cellulose region), 463–505 cm^−1^ (panel D—hemicellulose region), 833–878 cm^−1^ (panel E—pectin region), 905–952 cm^−1^ (panel F—lignin region), 1044–1059 cm^−1^ (panel G), 1073–1083 cm^−1^ (panel G), 1386–1413 cm^−1^ (panel H), 1570–1683 cm^−1^ (panel I—lignin region). The full spectrum is shown on panel J; Figure S2: PC1 score plotted against PC2 score. PCA was performed on the cortex spectra. The percentage of described variation is shown next to the axes; Figure S3: PC1 score plotted against PC3 score. PCA was performed on the cortex spectra. The percentage of described variation is shown next to the axes; Figure S4: PC1 score plotted against PC4 score. PCA was performed on the cortex spectra. The percentage of described variation is shown next to the axes; Figure S5: PC2 score plotted against PC3 score. PCA was performed on the cortex spectra. The percentage of described variation is shown next to the axes; Figure S6: PC2 score plotted against PC4 score. PCA was performed on the cortex spectra. The percentage of described variation is shown next to the axes; Figure S7: PC3 score plotted against PC4 score. PCA was performed on the cortex spectra. The percentage of described variation is shown next to the axes; Figure S8: PC1 score plotted against PC3 score. PCA was performed on the pith spectra. The percentage of described variation is shown next to the axes; Figure S9: PC1 score plotted against PC4 score. PCA was performed on the pith spectra. The percentage of described variation is shown next to the axes; Figure S10: PC2 score plotted against PC3 score. PCA was performed on the pith spectra. The percentage of described variation is shown next to the axes; Figure S11: PC2 score plotted against PC4 score. PCA was performed on the pith spectra. The percentage of described variation is shown next to the axes; Figure S12: PC3 score plotted against PC4 score. PCA was performed on the pith spectra. The percentage of described variation is shown next to the axes; Figure S13: One of the regions of the pith spectra that significantly contributed to the separation of the samples from different groups; Figure S14: Three regions of the pith spectra that significantly contributed to the separation of the samples from different groups; Figure S15: Two regions of the pith spectra that significantly contributed to the separation of the samples from different groups; Figure S16: One of the regions of the pith spectra that significantly contributed to the separation of the samples from different groups; Section S1: The comparison of the pith and cortex spectra.
