# Supplementary material for: MALDI MSI and Raman Spectroscopy Application in the Analysis of the Structural Components and Flavonoids in Brassica napus Stem
Source: Metabolites. 2023 May 25;13(6):687. doi: 10.3390/metabo13060687 (PMC10300850; doi:10.3390/metabo13060687)
Supplement: Supplementary file 1 [file metabolites-13-00687-s001.zip › metabolites-2384686-supplementary.pdf]

metabolites

Supplementary materials

1. Code S1: Python code adding names to the samples, performing vector normalization, performing principal components analysis, and creating loading plots.

```
import pandas as pd

import numpy as np

import matplotlib.pyplot as plt

from scipy.signal import find_peaks

from sklearn import decomposition

from sklearn.preprocessing import scale

import math

files=['K4_wew','K6_wew','K15_wew',

        'K60_8_wew','K60_11_wew','K60_15_wew',

        'RLN_5_wew','RLN_6_wew','RLN_9_wew']

names=['Control pith','Control pith','Control pith',

        'K60 pith','K60 pith','K60 pith',

        'RLN pith','RLN pith','RLN pith']

#

#

ext='.xlsx'

spectra_dict='' #path to spectra

Img_dict='' #path where images should be saved

sample_type='Rapeseed_raman_inner'

h_threshold=0.6

peak_threshold = 10 #the lenght of peak to be considered a peak in wavenumbers##


def take_closest(myList, myNumber):

    looked_number = min(myList, key=lambda x:abs(x-myNumber))

    return looked_number
```

```
def truncate(n, decimals=0):
    multiplier = 10 ** decimals
    return int(n * multiplier) / multiplier
def adding_name():
    df=pd.read_excel(spectra_dict+files[i]+ext)
    ss=df.T
    ss=ss[2:]
    j=0
    gr_name=[]
    while j<len(ss.columns):
        gr_name.append(names[i])
        j=j+1
    ss.columns=gr_name
    ss=ss.T
    return ss


def ann_loading_plot(PCx):#PC+number in ''
    int_range=np.sqrt((max(loading_matrix[PCx]))**2)+np.sqrt((min(loading_ma-
trix[PCx]))**2)
    Wav_range=np.sqrt((max(Wavenumbers))**2)-np.sqrt((min(Wavenumbers))**2)
    negpeaks=find_peaks(-loading_matrix[PCx],threshold=0.0000, dis-
tance=15,height=h_threshold, width=5) #change the parameters if You want to change
the peak finding alorithm
    pospeaks=find_peaks(loading_matrix[PCx],threshold=0.0000, dis-
tance=15,height=h_threshold, width=5) #change the parameters if You want to change
the peak finding alorithm
    peaks=np.concatenate((negpeaks[0],pospeaks[0])) #these peaks are only index of
peaks on x axis
    ab=pd.DataFrame(Wavenumbers[peaks]).to_excel(Img_dict+PCx+' peak_list.xlsx')
```

```
peak_range=[]
for line in Wavenumbers[peaks]:
    new_num=take_closest(thr_cross[PCx], line)
    peak_range.append(new_num)
    if line<new_num:
        add_i=thr_cross[thr_cross[PCx]==new_num].index.values[0]-1
    else:
        add_i=thr_cross[thr_cross[PCx]==new_num].index.values[0]+1
    peak_range.append(thr_cross[PCx][add_i])
peak_range=np.unique(peak_range)
peak_range=peak_range[~np.isnan(peak_range)]
new_peak_range=[]##
i=0##
while i<len(peak_range):##
    if (peak_range[i+1]-peak_range[i])>peak_threshold:##
        new_peak_range.append(peak_range[i])##
        new_peak_range.append(peak_range[i+1])##
    i=i+2##
peak_range=new_peak_range##
ab=pd.DataFrame(peak_range).to_excel(Img_dict+PCx+' range_list.xlsx')



fig, ax = plt.subplots()
ax.plot(Wavenumbers, loading_matrix[PCx])
ax.set_xlabel('Raman shift $(cm^{-1})$')
plt.title(PCx+' loading plot')
i=0
j=0
```

```
    z=0

    k=0

    while i<len(peak_range):

        t_end_i=Wavenumbers.get_loc(take_closest(Wavenumbers, peak_range[i+1]))
#gets indexes of begginning and end of the peaks over threshold

        t_beg_i=Wavenumbers.get_loc(take_closest(Wavenumbers, peak_range[i]))

        t_wave=Wavenumbers[t_beg_i:t_end_i]

        t_int=loading_matrix[PCx][t_beg_i:t_end_i]

        t_int.index=range(0,len(t_int))

        #if j>4:

        #       j=0

        if max(t_int)>0:

            y_pos= max(t_int) + 0.1*int_range + j*0.035*int_range #changes the upper
annotation position in y axis

            if j==4:

                y_pos= max(t_int) + 0.1*int_range + j*0.05*int_range

            if j==5:

                y_pos= max(t_int) + 0.1*int_range #+ j*0.04*int_range

            if j==6:

                y_pos= max(t_int) + 0.05*int_range #+ j*0.04*int_range

            j=j+1 #discard 'j' if the annotations do not cross each other



        else:

            y_pos= min(t_int) - 0.11*int_range #changes the lower annotations in y
axis

            y_pos= min(t_int) - 0.11*int_range - z*0.07*int_range

            if z==3:

                y_pos= min(t_int) - 0.09*int_range

            if z==4:
```

```
                y_pos= min(t_int) - 0.11*int_range - z*0.08*int_range

            z=z+1



            if z==6:

                z=1

        ax.plot(t_wave,t_int, color='r') #creates red part of the spectrum over/under the threshold

        if (len(t_int) % 2) == 0:

            x_pos=t_wave[int(len(t_int)/2)] #searches for the half x position of the range



        else:

            x_pos=t_wave[int((len(t_int)/2)-0.5)] #searches for the half x position of the range

        ann_len=len(str(math.ceil(Wavenumbers[t_beg_i])) +'-'+str(math.ceil(Wavenumbers[t_end_i])))

        annx_pos=x_pos-(ann_len*0.009*Wav_range) #changes the annotation position woth relation to the centre, length of annotation, and the range of trhe showed spectrum

        plt.text(annx_pos,y_pos, str(math.ceil(Wavenumbers[t_beg_i])) +'-'+str(math.ceil(Wavenumbers[t_end_i])))



    #       plt.annotate('' ,

    #                    xy=(x_pos,y_pos),

    #                    xytext=(x_pos,1.2*y_pos),

    #                    arrowprops=dict(facecolor='black',shrink=0.05)

    #                    )

        i=i+2

    plt.ylim(ymin=2*min(loading_matrix[PCx]),ymax=1.7*max(loading_matrix[PCx])) #determines the y range showed on the plot in relation to the range of the spectrum

    #plt.xlim(xmin=0.9*min(Wavenumbers),xmax=1.01*max(Wavenumbers))

    plt.savefig(Img_dict+PCx+' loading plot'+".jpg", dpi=300)
```

```
def PCA_scatter_plot(first_PC,second_PC):
    if first_PC=='PC1':
        axis1=0
    if first_PC=='PC2':
        axis1=1
    if first_PC=='PC3':
        axis1=2
    if first_PC=='PC4':
        axis1=3
    if first_PC=='PC5':
        axis1=4
    if second_PC=='PC1':
        axis2=0
    if second_PC=='PC2':
        axis2=1
    if second_PC=='PC3':
        axis2=2
    if second_PC=='PC4':
        axis2=3
    if second_PC=='PC5':
        axis2=4
    fig, ax = plt.subplots()
    a=0
    i=0
    for line in group_names:
        cur_nb_sam=number_of_samples.loc[line]
        temp_group=X[a:a+cur_nb_sam]
```

```
            ax.scatter(temp_group[:,axis1], temp_group[:,axis2],la-
bel=group_names[i],s=20)

            a=a+cur_nb_sam

            i=i+1

      ax.set_xlabel(first_PC+' ('+str(round((explained_var[axis1][0]),1))+'%)')

      ax.set_ylabel(second_PC+' ('+str(round((explained_var[axis2][0]),1))+'%)')

      plt.legend()

      plt.savefig(Img_dict+first_PC+' vs '+second_PC+' of '+sample_type+".jpg",dpi=300)



i=0

for line in files:

      if i==0:

            temp_df=adding_name()

            first_cols=temp_df.columns

      else:

            added_group=adding_name()

            added_group.columns=first_cols

            temp_df=pd.concat([temp_df,added_group])

      i=i+1



temp_df=temp_df.sort_index()



#vector normalization

sum_area=temp_df.sum(axis=1).values



i=0

while i<len(temp_df):

      row_multiplier=sum_area[0]/sum_area[i]
```

```
    one_row=pd.DataFrame(temp_df.iloc[i].multiply(row_multiplier)).T
    if i==0:
        norm_spec=one_row
    else:
        norm_spec=pd.concat([norm_spec,one_row])
    i=i+1

saving= norm_spec.to_excel(Img_dict+'normalised spectra.xlsx')
#PCA
Wavenumbers=norm_spec.columns

X = scale(norm_spec)
# apply PCA
pca = decomposition.PCA(n_components=5)
X = pca.fit_transform(X)

loadings = pca.components_.T * np.sqrt(pca.explained_variance_)

loading_matrix = pd.DataFrame(loadings, columns=['PC1', 'PC2','PC3','PC4','PC5'])

explained_var=pd.DataFrame(pca.explained_variance_ratio_*100)
explained_var=explained_var.values.tolist()
group_names=norm_spec.index.unique().values
number_of_samples=norm_spec.pivot_table(index = norm_spec.index, aggfunc
='size')

#threshold crossing
```

```
for line in loading_matrix.columns:
    curr_over_thr=[]
    i=1
    while i<len(loading_matrix[line]):
        if i==1 and loading_matrix[line][0]>h_threshold:
            curr_over_thr.append(Wavenumbers[0])
        if i==1 and loading_matrix[line][0]<-h_threshold:
            curr_over_thr.append(Wavenumbers[0])
        if loading_matrix[line][i-1] < h_threshold and loading_matrix[line][i] > h_threshold:
            curr_over_thr.append(Wavenumbers[i])
        if loading_matrix[line][i-1] > h_threshold and loading_matrix[line][i] < h_threshold:
            curr_over_thr.append(Wavenumbers[i])
        if loading_matrix[line][i-1] < -h_threshold and loading_matrix[line][i] > -h_threshold:
            curr_over_thr.append(Wavenumbers[i])
        if loading_matrix[line][i-1] > -h_threshold and loading_matrix[line][i] < -h_threshold:
            curr_over_thr.append(Wavenumbers[i])
        if i==(len(loading_matrix[line])-1) and loading_matrix[line][i] > h_threshold:
            curr_over_thr.append(Wavenumbers[i])
        if i==(len(loading_matrix[line])-1) and loading_matrix[line][i] < -h_threshold:
            curr_over_thr.append(Wavenumbers[i])
        i=i+1
    if line==loading_matrix.columns[0]:
        thr_cross=pd.DataFrame({line:curr_over_thr}) #creates the matrix with the values of x that cross the threshold with the peaks inside
    else:
        thr_cross=pd.concat([thr_cross,pd.DataFrame({line:curr_over_thr})],axis=1)
```

```
PCA_scatter_plot('PC1', 'PC2')

#PCA_scatter_plot('PC1', 'PC3')

#PCA_scatter_plot('PC1', 'PC4')

#PCA_scatter_plot('PC1', 'PC5')

#PCA_scatter_plot('PC2', 'PC3')

#PCA_scatter_plot('PC2', 'PC4')

#PCA_scatter_plot('PC2', 'PC5')

#PCA_scatter_plot('PC3', 'PC4')

#PCA_scatter_plot('PC3', 'PC5')

#PCA_scatter_plot('PC4', 'PC5')



#ann_loading_plot('PC1')

#ann_loading_plot('PC2')
```

2. Code S2: Python code creating the spectra with 95% confidence interval for mean from the whole spectral range and from the parts of the spectra that cross the threshold

```
"""

Suitable for low to high x scale

"""



import seaborn as sns

import pandas as pd

spectra_dict=''#path, name and extention of the spectra

Img_dict='' #path where images should be saved

peak_range_dict='' #path, name and extention of the file with peak range crossing the threshold

sample_type='Rapeseed_raman_inner'
```

```
def take_closest(myList, myNumber):
    looked_number = min(myList, key=lambda x:abs(x-myNumber))
    return looked_number


def truncate(n, decimals=0):
    multiplier = 10 ** decimals
    return int(n * multiplier) / multiplier



df=pd.read_excel(spectra_dict)
ef=pd.read_excel(peak_range_dict)[0]


df=df.set_index(df.columns[0])
group_names=df.index.unique().values
number_of_samples=df.pivot_table(index = df.index, aggfunc ='size')


all_names=[]
i=0
for line in group_names:
    cur_nb_sam=number_of_samples.loc[line]
    j=0
    while j<cur_nb_sam:
        all_names.append(group_names[i])
        j=j+1
    i=i+1



temp_df=df.T
```

```
Wavenumbers=temp_df.index
raveled_df=temp_df.values.ravel().tolist()


raveled_wav=[]
for line in Wavenumbers:
    i=0
    while i<len(temp_df.columns):
        raveled_wav.append(line)
        i=i+1


raveled_names=[]
for line in Wavenumbers:
    raveled_names.extend(temp_df.columns.values.ravel().tolist())


ab=pd.DataFrame((raveled_names, raveled_wav, raveled_df)).T
ab.columns=['Sample name','Raman shift $(cm^{-1})$','Intensity']


figure=sns.relplot( #shows 95% confidence interval for mean
    data=ab, kind="line",
    x="Raman shift $(cm^{-1})$", y="Intensity",
    hue="Sample name",aspect=1.39,
    )
figure.savefig(Img_dict+'Whole spectrum of '+ sample_type+'.jpg',dpi=300)
def plot_range_with_peak(PCx, showed_range):
    def choose_highest (i_pos):
        i=0
        for line in xydata:
            if i==0:
```

```
                max_y=xydata[i][i_pos,1]
            else:
                if len(xydata[i])>0:
                    if max_y<xydata[i][i_pos,1]:
                        max_y=xydata[i][i_pos,1]
            i=i+1
        return max_y
    def gib_int_range():
        i=0
        for line in xydata:
            if i==0:
                max_in_range=max(xydata[i][:,1])
            else:
                if len(xydata[i])>0:
                    if max_in_range<max(xydata[i][:,1]):
                        max_in_range=max(xydata[i][:,1])
            i=i+1
        i=0
        for line in xydata:
            if i==0:
                min_in_range=min(xydata[i][:,1])
            else:
                if len(xydata[i])>0:
                    if min_in_range>min(xydata[i][:,1]):
                        min_in_range=min(xydata[i][:,1])
            i=i+1
        int_range=max_in_range-min_in_range
        return int_range
```

```
def gib_wav_range():
    range_wav=max(xydata[0][:,0])-min(xydata[0][:,0])
    return range_wav



range_beg= ef[j]
range_end= ef[j+1]
ranges=[range_beg,range_end]
z=j+1
added_index=0
while z<len(ef)-1:
    if ef[z+2]-ef[j]<showed_range:
        ranges.append(ef[z+1])
        ranges.append(ef[z+2])
        added_index=added_index+2
    z=z+2



temp_wav=Wavenumbers.tolist()
peak_pos=(ranges[len(ranges)-1]+ranges[0])/2

beg_iorig=temp_wav.index(take_closest(temp_wav, peak_pos-showed_range))
end_iorig=temp_wav.index(take_closest(temp_wav, peak_pos+showed_range))

beg_i=beg_iorig*len(temp_df.columns)
end_i=(end_iorig+1)*len(temp_df.columns)

spectra_to_show=ab[beg_i:end_i]
```

```
figure=sns.relplot( #shows 95% confidence interval for mean
        data=spectra_to_show, kind="line",
        x="Raman shift $(cm^{-1})$", y="Intensity",
        hue="Sample name",aspect=1.39,
        )
xydata = []
for ax in figure.axes.flat:
        for li in ax.lines:
                xydata.append(li.get_xydata())

poses_i=[]
for line in ranges:
        pos_i=xydata[0][:,0].tolist().index(take_closest(xydata[0][:,0], line))
        poses_i.append(pos_i)

int_range=gib_int_range()
wav_range=gib_wav_range()



i=0
while i<len(poses_i):
        ann_len=len(str(truncate(ranges[i],1))+'-'+str(truncate(ranges[i+1],1)))
        ax.text(((ranges[i]+ranges[i+1])/2)-(ann_len*0.008*wav_range),
max([choose_highest(poses_i[i]),choose_highest(poses_i[i+1])])+0.03*int_range,
str(truncate(ranges[i],1))+'-'+str(truncate(ranges[i+1],1)),weight='bold')
        ax.annotate('',
```

```
                        xy=(ranges[i], choose_highest(poses_i[i])),
                        xytext=(ranges[i+1], choose_highest(poses_i[i+1])),
                        xycoords='data',
                        arrowprops=dict(arrowstyle="<|-
|>,head_length=0.75,head_width=0.3", facecolor='black',linewidth=4))
            i=i+2 #
      figure.savefig(Img_dict+PCx+' peak at '+ str(truncate(peak_pos,1))+'.jpg',dpi=300)
      return added_index


j=0
while j<len(ef):
      add_i=plot_range_with_peak('PC1',100)
      j=j+2+add_i
```

3. Figures S1-S5

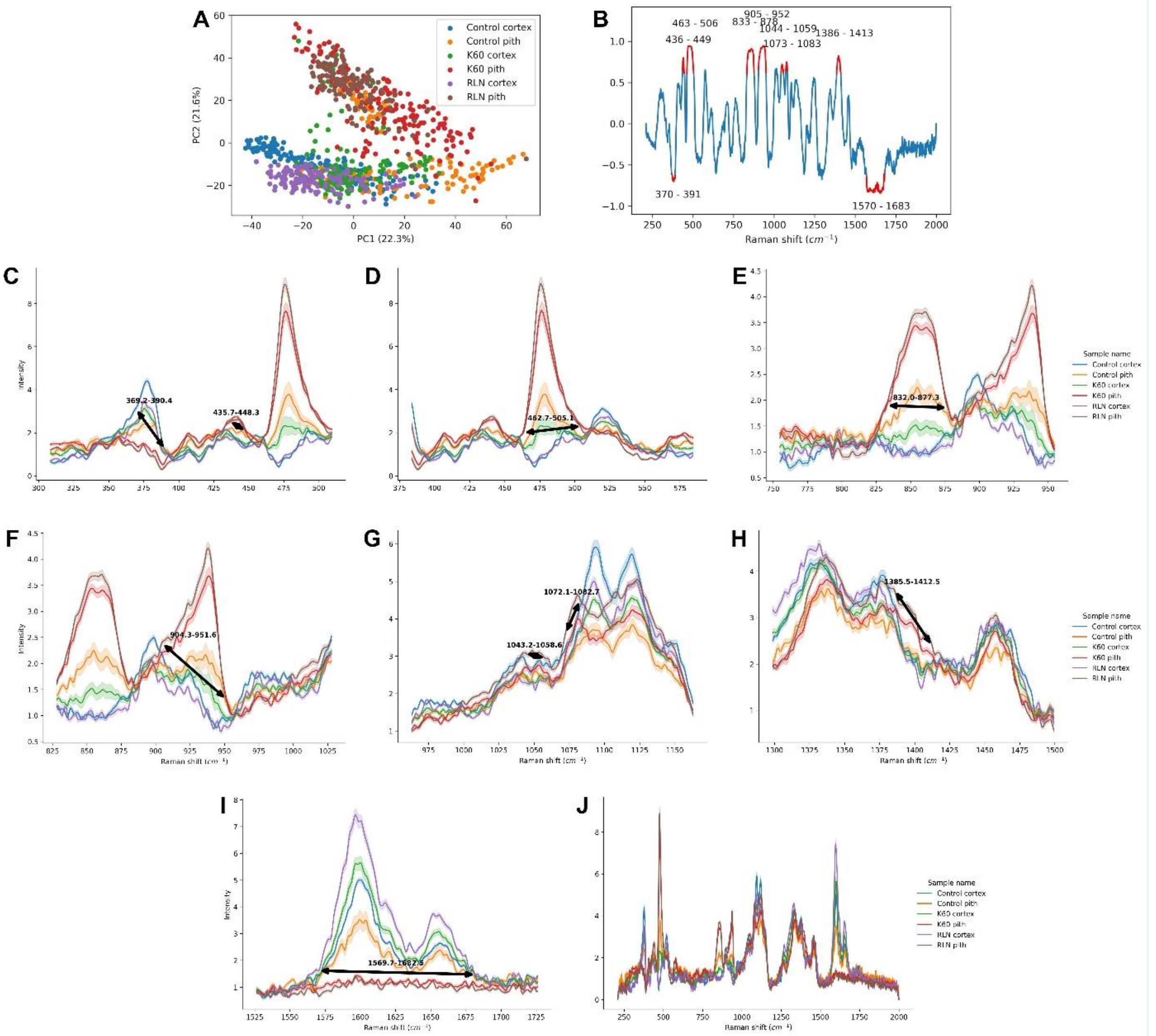

Figure S1. PCA, loading plot and mean spectra (with 95% confidence interval) of regions that were above the 0.6 (or below -0.6) threshold in the loading plot. In the PCA PC1 score is plotted against the PC2 score (A) and the percentage of described variation is shown next to the axes. The loading plot (B) shows the PC2 loading plot with the regions crossing the threshold of above 0.6 or below -0.6, which are considered significant. The regions that cross the threshold are: 370-391 cm^{-1} (spectra of this region showed on panel C – cellulose region), 436-448 cm^{-1} (panel C – cellulose region), 463-505 cm^{-1} (panel D – hemicellulose region), 833-878 cm^{-1} (panel E – pectin region), 905-952 cm^{-1} (panel F – lignin region), 1044-1059 cm^{-1} (panel G), 1073-1083 cm^{-1} (panel G), 1386-1413 cm^{-1} (panel H), 1570-1683 cm^{-1} (panel I – lignin region). The full spectrum is shown on panel J.

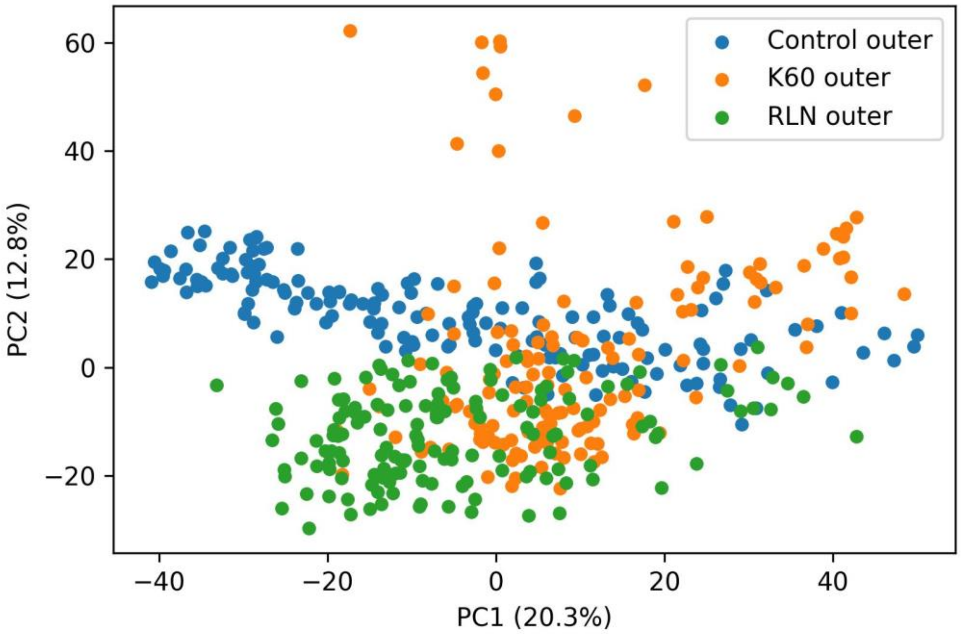

Figure S2. PC1 score plotted against PC2 score. PCA was performed on the cortex spectra. The percentage of described variation is shown next to the axes.

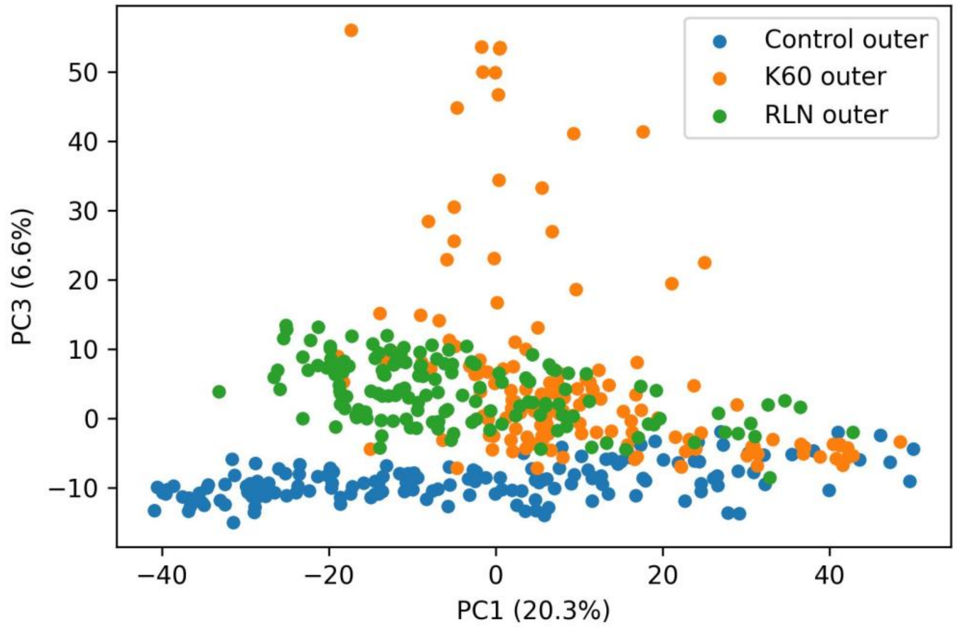

Figure S3. PC1 score plotted against PC3 score. PCA was performed on the cortex spectra. The percentage of described variation is shown next to the axes.

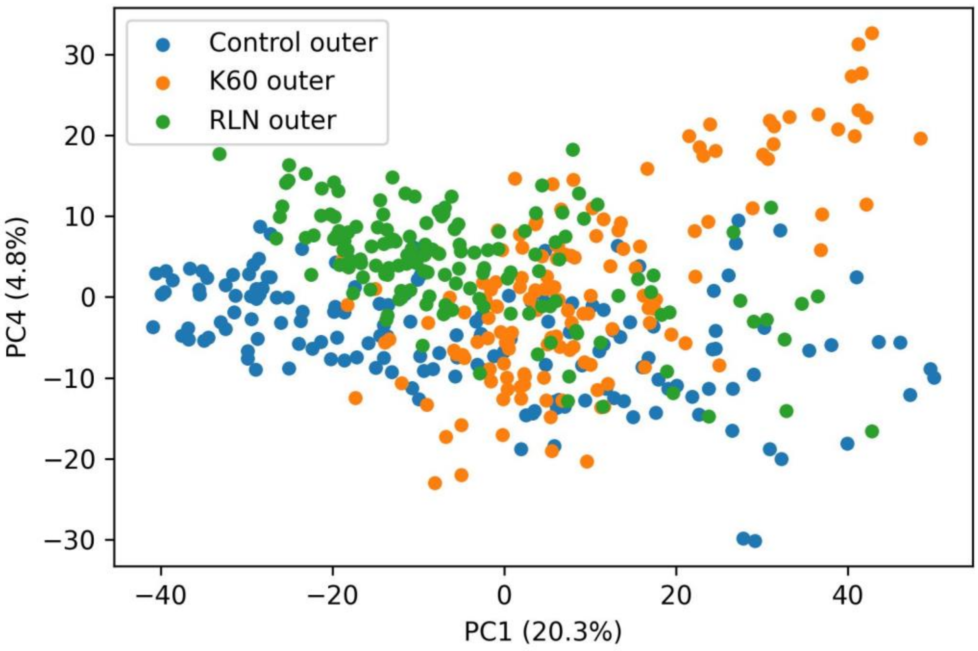

Figure S4. PC1 score plotted against PC4 score. PCA was performed on the cortex spectra. The percentage of described variation is shown next to the axes.

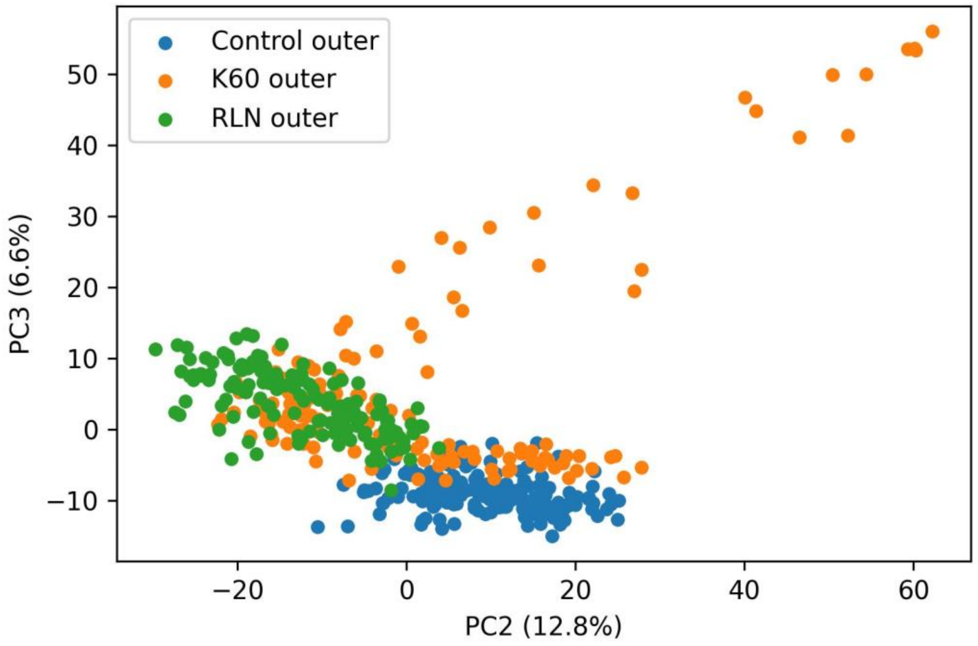

Figure S5. PC2 score plotted against PC3 score. PCA was performed on the cortex spectra. The percentage of described variation is shown next to the axes.

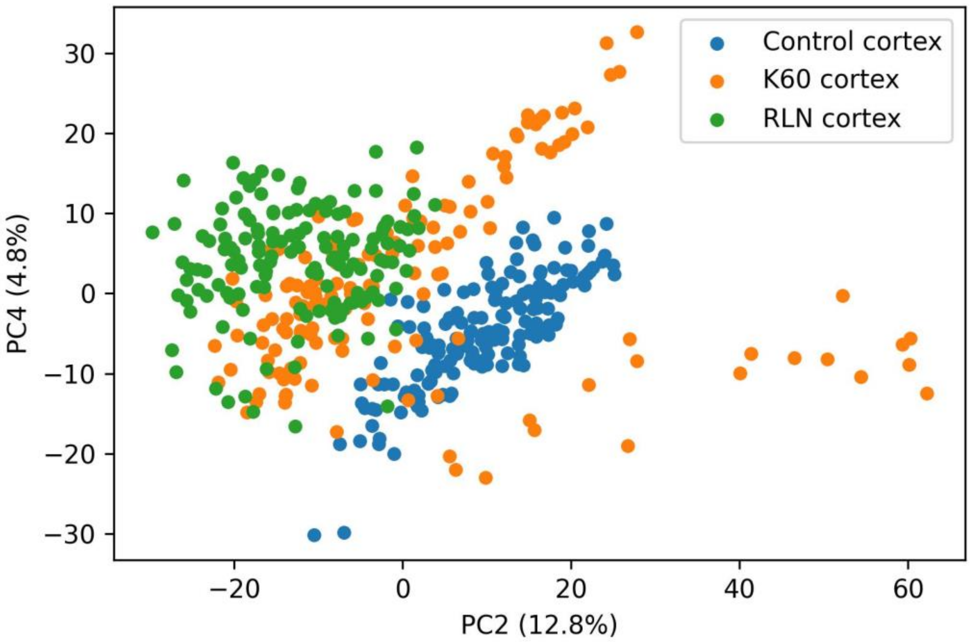

Figure S6. PC2 score plotted against PC4 score. PCA was performed on the cortex spectra. The percentage of described variation is shown next to the axes.

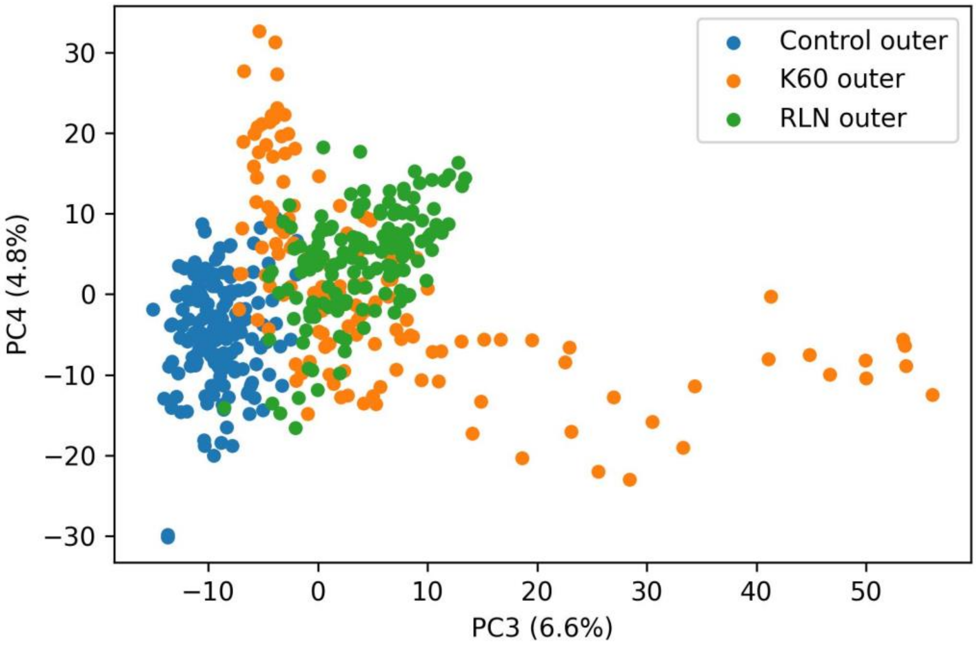

Figure S7. PC3 score plotted against PC4 score. PCA was performed on the cortex spectra. The percentage of described variation is shown next to the axes.

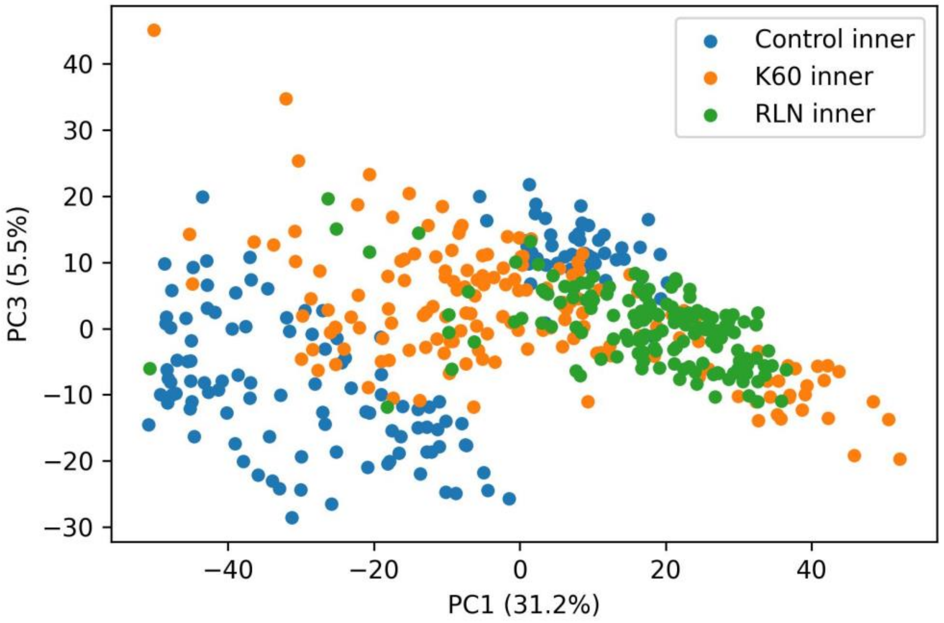

Figure S8. PC1 score plotted against PC3 score. PCA was performed on the pith spectra. The percentage of described variation is shown next to the axes.

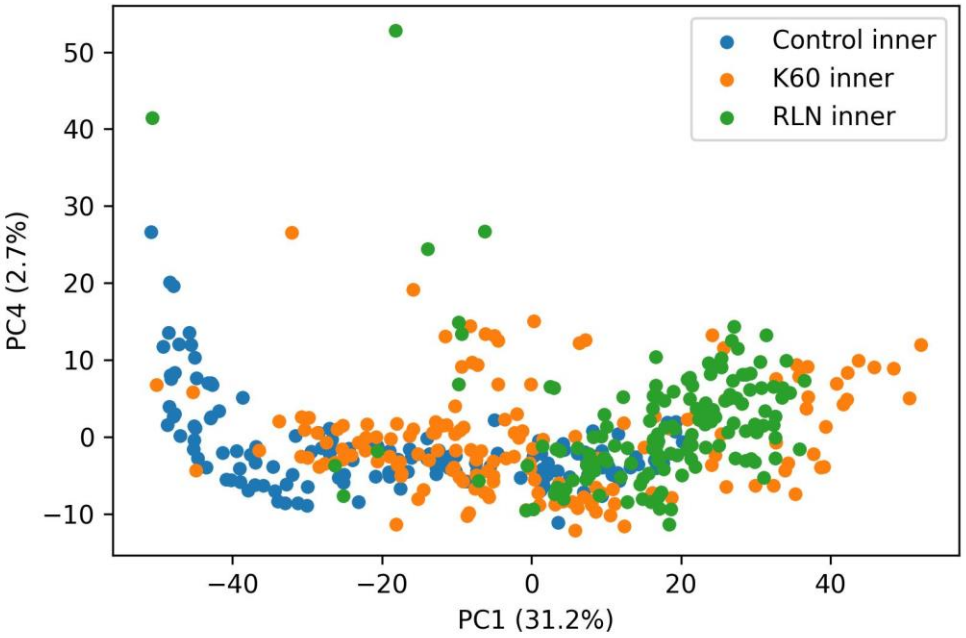

Figure S9. PC1 score plotted against PC4 score. PCA was performed on the pith spectra. The percentage of described variation is shown next to the axes.

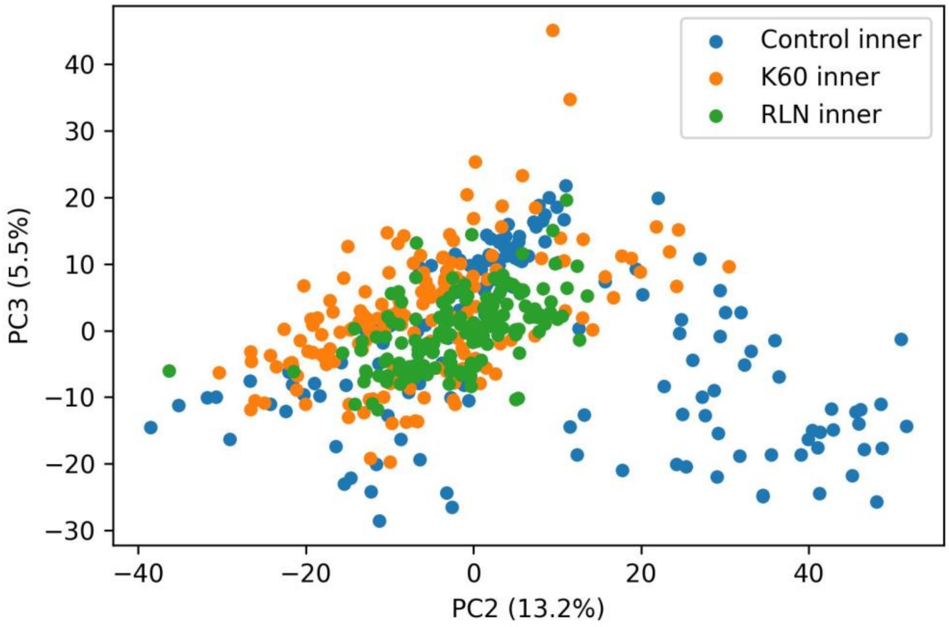

Figure S10. PC2 score plotted against PC3 score. PCA was performed on the pith spectra. The percentage of described variation is shown next to the axes.

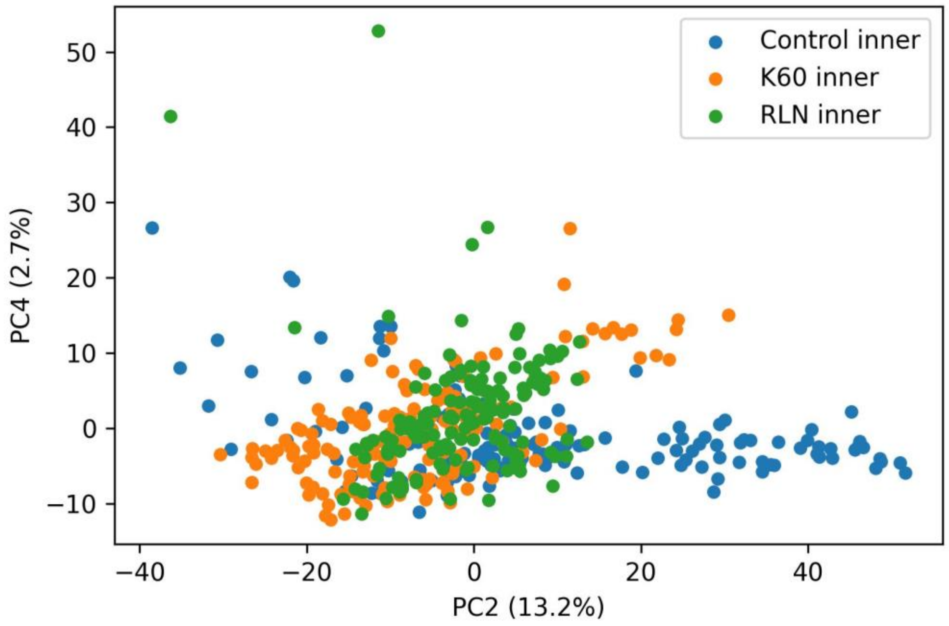

Figure S11. PC2 score plotted against PC4 score. PCA was performed on the pith spectra. The percentage of described variation is shown next to the axes.

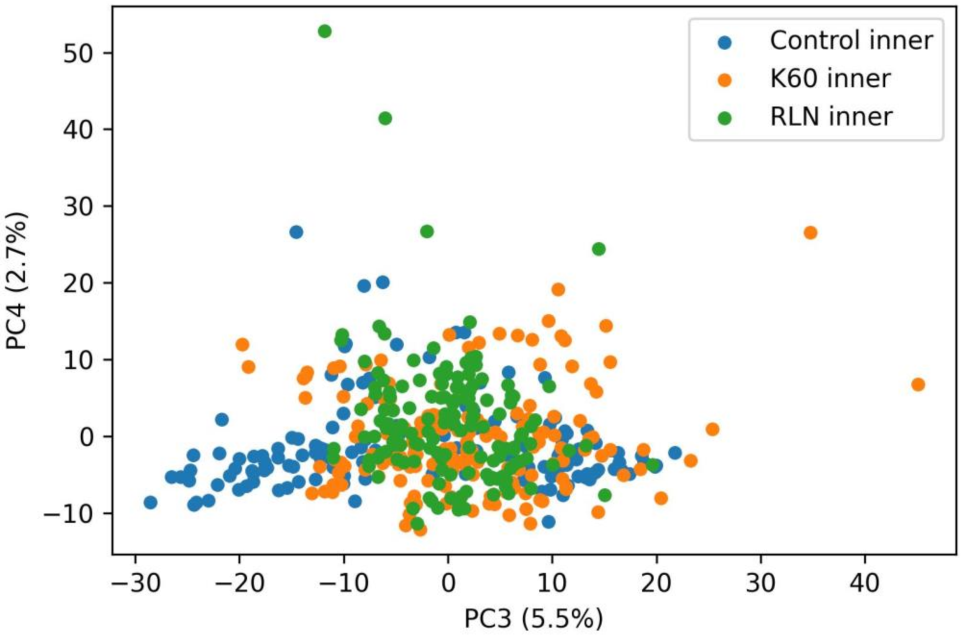

Figure S12. PC3 score plotted against PC4 score. PCA was performed on the pith spectra. The percentage of described variation is shown next to the axes.

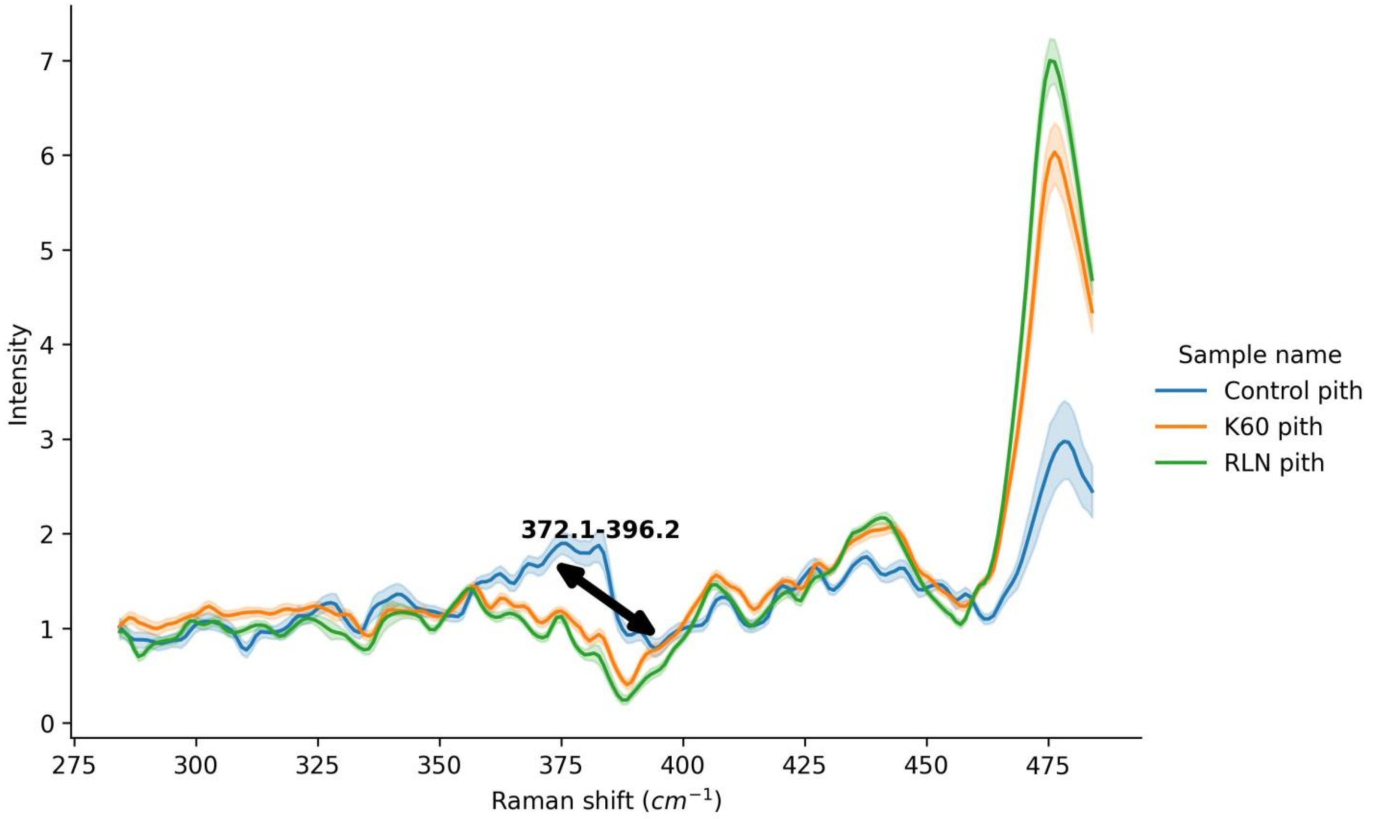

Figure S13. One of the regions of the pith spectra that significantly contributed to the separation of the samples from different groups.

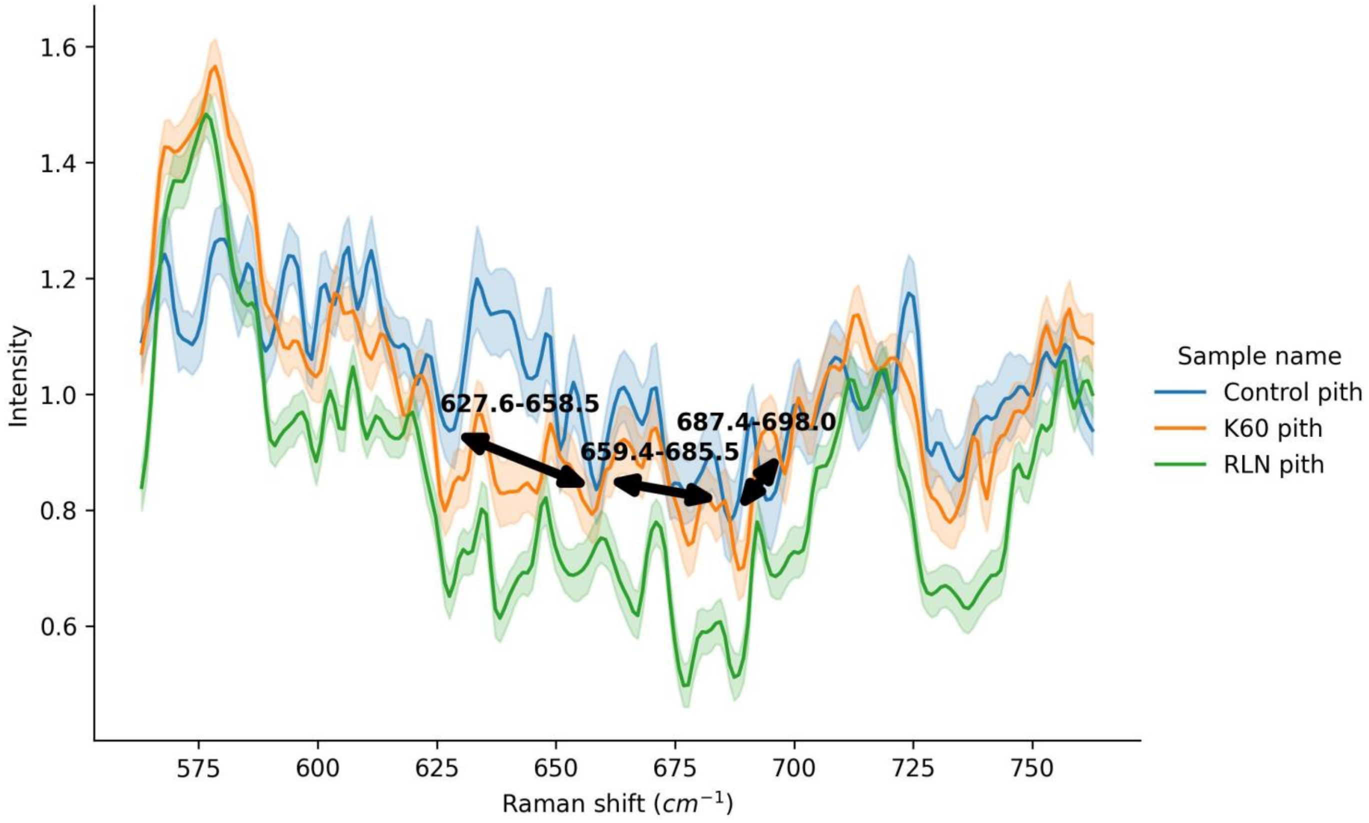

Figure S14. Three regions of the pith spectra that significantly contributed to the separation of the samples from different groups.

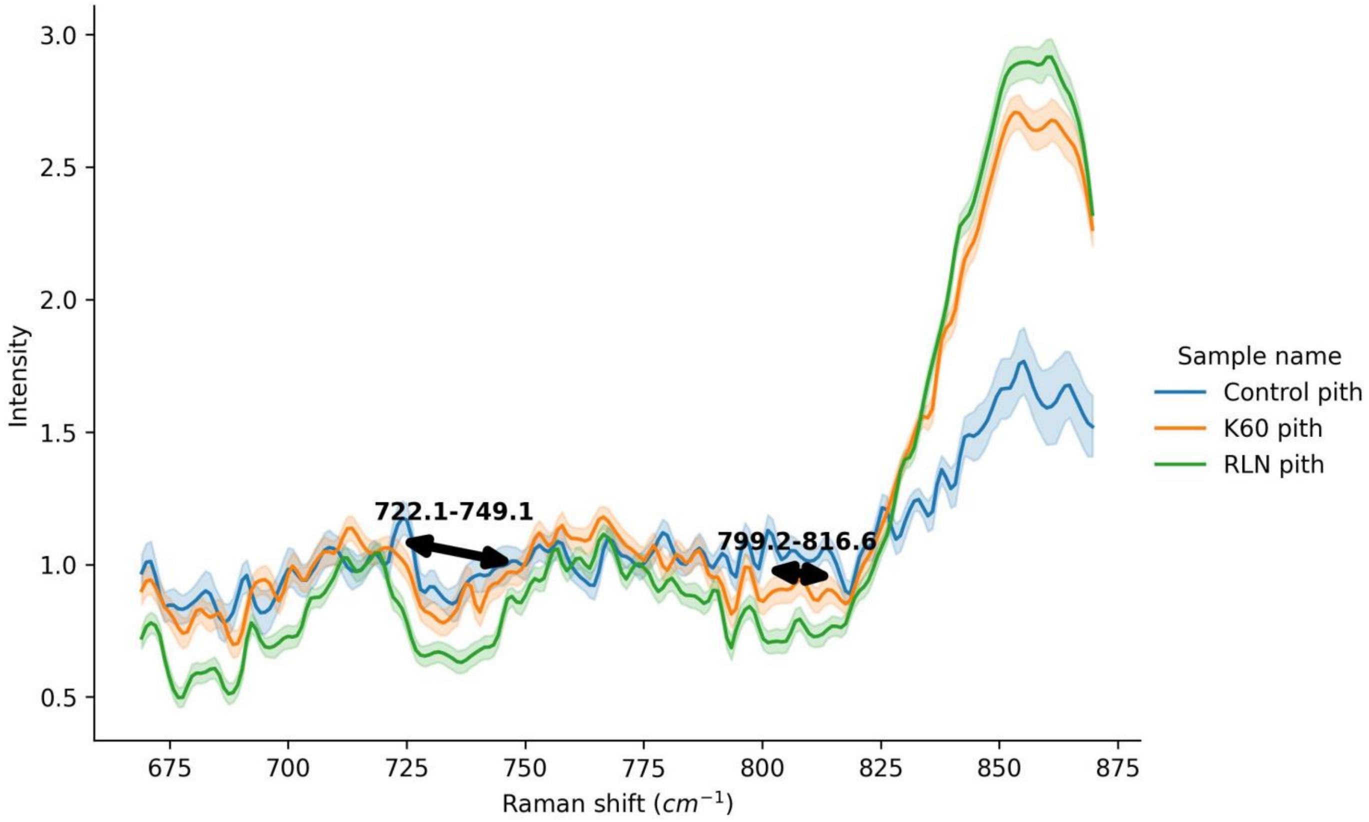

Figure S15. Two regions of the pith spectra that significantly contributed to the separation of the samples from different groups.

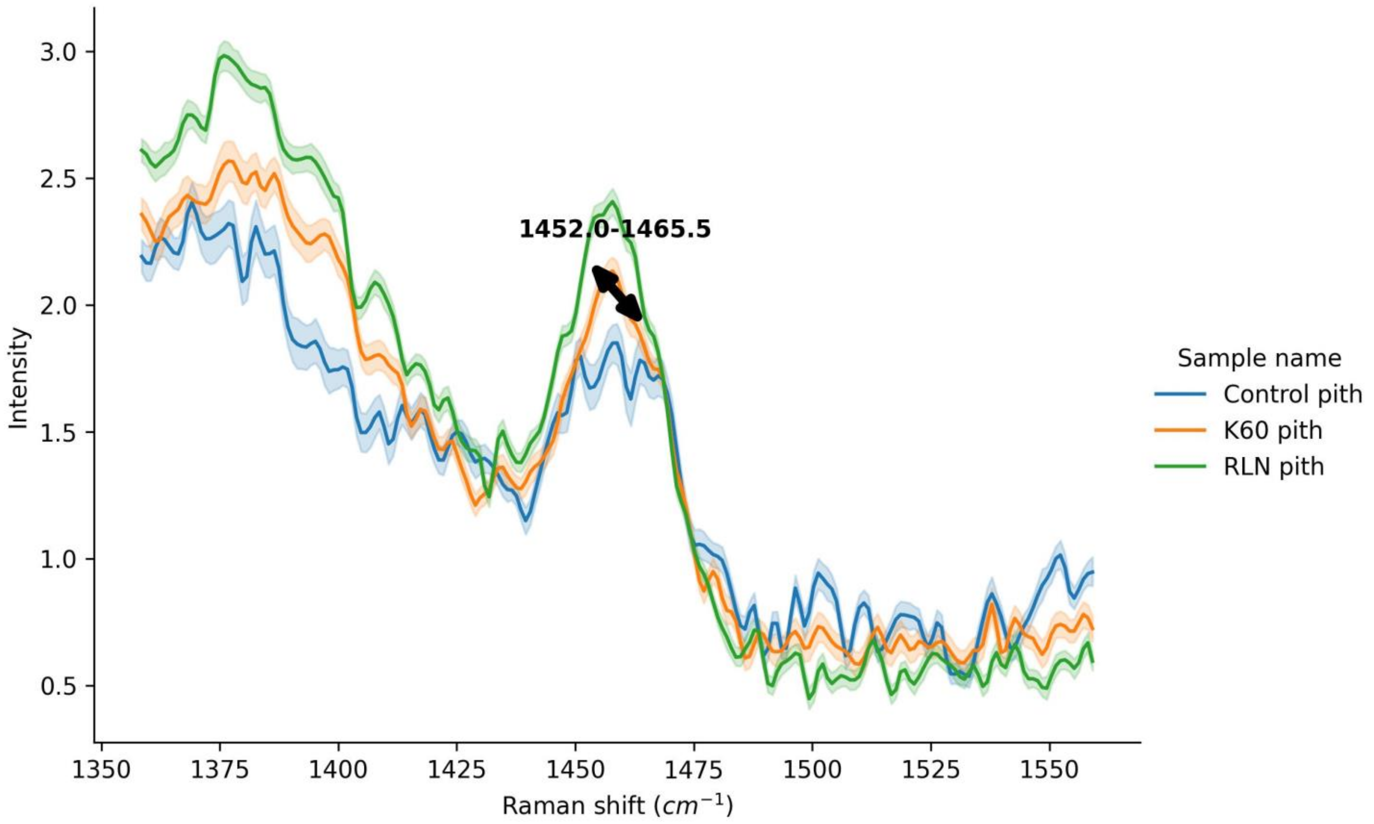

Figure S16. One of the regions of the pith spectra that significantly contributed to the separation of the samples from different groups.

4. Section S1. The comparison of the pith and cortex spectra

For the differentiation between the pith and cortex spectra plotting of the PC1 against PC2 score proved to give the best results (Fig. S1.A). Most of the separation occurred due to PC2 except the spectra of the control pith sample. Some of control pith spectra are mixed with the cortex spectra and some of it is separated due to PC1 score, however its spectra is the most similar to those of cortex. Because of the fact that the most separation occurred due to the PC2, the loading plot of PC2 (Fig. S1.B.) will be interpreted.

First region that significantly separated pith from the cortex in PC2 was 370-391 cm^{-1} – a band of cellulose. This band has the highest area in the cortex samples (Fig. S1.C.). The next significantly separated band was also of cellulose origin, but due to the CCO ring stretching in the region of 436-448 cm^{-1}. This band has higher area in the pith samples, however the difference was lower than in the previous example (Fig. S1.C.). Highly separating region and the one with high area difference is a 463-505 cm^{-1} region, which is characteristic for composite bending vibrations involving the C6 position of hemicellulose. It presented much higher area in the pith samples (Fig. S1.D.). Similar changes were seen in the band between 833-878 cm^{-1} and between 905-952 cm^{-1} due to the COC skeletal vibrations of pectins and due to the CCH wagging of lignin respectively (Fig. S1.E., Fig. S1.F. respectively). The next regions separating the spectra significantly were 1044-1059 cm^{-1},

1073-1083 cm^{-1} (Fig. S1.G.) and 1386-1413 cm^{-1} (Fig. S1.H.). The first one did not show clear differences between the groups, the two latter seem to have slightly higher area in the pith samples, however the differences are to slight to draw the conclusions. The last significantly separating band of PC2 loading plot is the band in the 1570-1683 cm^{-1}, which covers symmetric aryl ring stretching, ring C=C stretching of coniferyl alcohol and C=O stretching of coniferaldehyde of lignin. This band has significantly higher area in the cortex spectra (Fig. S1.I.). The whole spectra are presented on Figure S1.J.

Overall cortex seem to have higher concentration of cellulose and lower of hemicellulose, pectins and lignins than pith.

While most of the studies showing the differences between the pith and the cortex focuses on the monocotyledon plants, whereas both the amount of cellulose as well as lignin is higher in the cortex than in pith [35,36],there are some studies showing the differences between these structures in dicotyledonous plants [37,38]. These studies show the higher lignification of the pith than the cortex, which is in line with our findings. Those studies however do not emphasize on the concentration of hemicellulose and pectins, which we found to have higher concentration in the pith.
